# Urinary hepcidin level as an early predictor of iron deficiency in children: A case control study

**DOI:** 10.1186/1824-7288-37-37

**Published:** 2011-08-11

**Authors:** Mohammed Sanad, Amal F Gharib

**Affiliations:** 1 Department of Pediatrics, Faculty of Medicine, Zagazig University, Egypt; 2 Department of Biochemistry, Faculty of Medicine, Zagazig University, Egypt

## Abstract

**Background:**

The ideal screening test would be capable of identifying iron deficiency in the absence of anemia. We tried to detect role of urinary hepcidin-25 level in early prediction of iron deficiency in children.

**Methods:**

This is a case control study performed on 100 children in Hematology Unit of Pediatric Department, Zagazig University Hospital, Egypt. Our study included 25 cases of iron deficiency (ID) stage-1 (iron depletion), 25 cases ID stage-2 (iron-deficient erythropoiesis), 25 cases ID stage-3 (iron deficiency anemia) and 25 healthy children as a control group. Estimation of iron status parameters was done. Urinary hepcidin-25 level was detected.

**Results:**

Urinary hepcidin-25 level was significantly lower in all stages of iron deficiency than in control group, more significant reduction in its level was observed with the progress in severity of iron deficiency. Urinary hepcidin showed significant positive correlation with hemoglobin, mean corpuscular volume, hematocrit value, serum iron and ferritin and transferrin saturation. In contrary, it showed significant negative correlation with serum transferrin and total iron binding capacity.

Urinary hepcidin at cutoff point ≤0.94 nmol/mmol Cr could Predict ID stage-1 with sensitivity 88% and specificity 88%. Cutoff point ≤0.42 nmol/mmol Cr could predict ID stage-2 with sensitivity 96% and specificity 92%. Cutoff point ≤0.08 nmol/mmol Cr could Predict ID stage-3 with Sensitivity 96% and specificity 100%.

**Conclusions:**

We can conclude that detection of urinary hepcidin-25 level was a simple and non invasive test and could predict iron deficiency very early, before appearance of hematological affections.

## Background

Iron deficiency anemia (IDA) affects approximately 30% of the world's population with more prevalent in children and neonates [1]. IDA in infants and toddlers is associated with long-lasting diminished mental, motor and behavioral effects [2]. Treatment with iron leads to subsequent complete resolution of the anemia and the iron deficiency (ID). Yet, it does not correct all of the behavior effects [3]. Furthermore, the lower mental and motor test scores associated with iron deficiency anemia persist [2,4].

Three stages of ID may be recognized. The first is substantial reduction in normal iron stores (iron depletion) [5]. This first stage of ID is often characterized by low serum ferritin levels [6]. The second stage of ID is a state of iron-deficient erythropoiesis. In this stage, there is a shortage of iron available to the erythroid precursors in the bone marrow for hemoglobin (Hb) synthesis [7]. The second stage may be characterized by abnormalities in particular iron parameters, including low transferrin saturation (Tsat) levels and elevations in free erythrocyte zinc protoporphyrin (ZnPP) [6]. The third and most severe degree of ID involves overt microcytic anemia due to insufficient iron stores to maintain red blood cell synthesis, leading to anemia [7]. IDA is characterized by a significant reduction in hemoglobin level and a decrease in mean corpuscular volume (MCV) [6].

Additional laboratory findings in IDA include elevated total iron-binding capacity (TIBC), low Tsat and low serum iron level [2]. Serum ferritin is the best laboratory test for the diagnosis of iron deficiency as it may decrease before a decrease in serum iron level is detected [8]. It is noteworthy that ferritin level increases with age. Besides, it is an acute-phase reactant that may be falsely elevated in the setting of chronic inflammation, infection, malignancy and chronic renal failure [2].

The erythrocyte zinc protoporphyrin assay (ZnPP) is another laboratory screening test which is used to assess iron status. In IDA, there is an increase in zinc transport across the intestinal membrane to replace the missing iron in the formation of the protoporphyrin ring [9]. ZnPP assay is a sensitive test; however its specificity may be limited because ZnPP increases in inflammation, lead poisoning, anemia of chronic diseases (ACD) and hemoglobinopathies [10].

Hepcidin is the principal iron-regulatory hormone that mediates the homeostasis of extracellular iron concentrations [11,12]. Hepcidin is initially synthesized as an 84-amino acid preprohepcidin then it is processed in hepatocytes by a signal peptidase and the prohormone convertase furin [13] to its bioactive form, 25-amino acid peptide [14,15]. Hepcidin is produced by hepatocytes and is rapidly cleared from the circulation [14]. Urinary hepcidin levels correlate well with hepatic hepcidin mRNA [16]. Three hepcidin isoforms (hepcidin-20, -22 and -25) are excreted in urine. Hepcidin-25 and -20 are also found in serum [14]. Hepcidin-25 is the only isoform which has a dominant role in iron regulation [17]. Hepcidin acts by regulating iron influx into plasma from tissues engaged in iron storage or transport: duodenal enterocytes that absorb dietary iron, hepatocytes that store iron, and macrophages that recycle iron from senescent erythrocytes. At the molecular level, hepcidin binds to the sole known cellular iron efflux channel, ferroportin, and induces its internalization and lysosomal degradation by mechanisms similar to those that inactivate other more conventional membrane receptors [18]. N-terminally truncated breakdown products of hepcidin are detectable in plasma [19] and urine [14,15] but show impaired ability to internalize ferroportin [20]. Hepcidin synthesis is physiologically increased by elevated plasma iron concentration [21,22], decreased by erythropoietic activity [23] and pathologically increased by inflammation [21,24]. Hepcidin excess plays the major role in anemia of inflammation [25]and iron-resistant iron-deficiency anemia [15,26]. At the opposite extreme, hepcidin deficiency is the cause of iron overload in most hereditary hemochromatoses [27] and contributes to iron overload in β-thalassemia and other iron-loading anemia's [28]. Urine testing is chosen in preference to serum assays as (I) it is less affected by diurnal variation, and (II) the non-invasive nature of sampling [17].

### Aim of the work

To detect role of urinary hepcidin-25 level in early prediction of iron deficiency in children.

## Methods

This was a case control study performed in Zagazig University Children Hospital and Outpatient Clinics in the same Hospital from October 2009 to October 2010. Informed parental consent was obtained to be eligible for enrollment into the study. The study was done according to the rules of the Local Ethics Committee of Faculty of Medicine, Zagazig University. The study included 75 children presented with ID and 25 healthy children of comparable age and sex as a control group. Children were classified as follows:

### ID stage-1

25 children presented with normal iron parameters and hematological parameters except low serum ferritin level (≤ 20 ng/ml) [6,7,29].

### ID stage-2

25 children presented with low serum ferritin (< 12 ng/ml), low serum Tsat < 16% and increased ZnPP (> 100 ug/dl). Hemoglobin level, MCV and mean corpuscular hemoglobin concentration (MCHC) were normal for age [6,29].

### ID stage-3

25 children presented with low serum ferritin (< 12 ng/ml), low Tsat (< 16%), increased ZnPP (> 100 ug/dl) and picture of microcytic hypochromic anemia (low hemoglobin level, low MCV and low MCHC) [6,29].

### Control group

25 healthy children presented with normal iron parameters [29], as serum ferritin level was > 20 ng/ml, ZnPP level was ≤30 ug/dl and Tsat was > 35%. They were selected from those who attended Pediatric Department for preoperative evaluation for elective surgery (eg Hernia, hydrocele and undescended testis).

Samples were collected relatively during a narrow time period (late morning to early afternoon) and iron levels were not interpreted in isolation, rather combined with other iron parameters.

As ferritin, an acute phase reactant, may be elevated during co-morbid infection(s), we excluded any patient presented with any evidences of infections or inflammations [17]. Children were excluded if there were evidences of renal function abnormalities, liver function abnormalities or history of iron therapy in the previous three months.

All patients and controls included were subjected to proper history taking, thorough clinical examination. Laboratory investigations were done for all studied children and included urinalysis, stool analysis, complete blood count (including blood indices), ESR, C-reactive protein, liver function and kidney function tests. Iron parameters (iron, ferritin, transferrin, MCHC, ZnPP, TIBC and Tsat), creatinine level in urine and urine hepcidin-25 level were estimated.

**Free erythrocyte zinc protoporphyrin **was estimated in whole blood using a fluorometric method, in which porphyrins and heme components were extracted from whole blood into an ethyl acetate-acetic acid mixture. Porphyrins were then separated from heme by back-extraction into a hydrochloric acid solution, and were quantitatively determined by molecular fluorometry using a spectrofluorometer calibrated with protoporphyrin IX (PPIX) standard solutions.

### Creatinine analysis in urine

Urinary creatinine concentrations were measured by the Jaffe reaction using the Creatinine Parameter Assay (R&D Systems, Minneapolis, MN).

### Urinary hepcidin analysis

Freshly voided urine samples were collected and 10 mL aliquots were centrifuged at 5000 rpm for 5 minutes and frozen at -80°C for batch analyses at the Department of Biochemistry, according to the manufacturer's protocol; 96-well plates were coated with the antibody to human hepcidin and incubated with 100 μL (standard samples) or 200 μL (samples with very low concentration of hepcidin) of 1:10 dilution of urine in Tris-buffered saline containing 0.05% Tween-20 (TBS-Tween 20), with 10 ng/mL of biotinylated hepcidin-25 (Intrinsic LifeSciences, La Jolla, CA) added as the tracer. Standard curves were prepared by serial 2-fold dilution of synthetic hepcidin (Bachem Biosciences, King of Prussia, PA) 4000 ng/mL in TBS-Tween 20 buffer containing the tracer. The integrity and bioactivity of synthetic hepcidin and biotinylated hepcidin were verified by spectrometry and by bioassay [20]. After washing, the assay was developed with streptavidin-peroxidase and tetramethyl benzidine. The enzymatic reaction was stopped by sulfuric acid, and the plate was read at 450 nm on a DTX 880 microplate reader (Beckman Coulter, Fullerton, CA). Standard curves were fitted with 12-point fit using GraphPad Prism software (GraphPad Software, San Diego, CA). The fitted curve was then used to convert sample absorbance readings to hepcidin concentrations. The intra-assay coefficient of variation (CV) was ranged from 5-18% and the inter-assay CV was ranged from 3-14%. The lower limit of detection was 0.07 nmol. To account for the variable dilution of urine samples, urine hepcidin concentrations were normalized to urine creatinine (Cr).

### Statistical analysis

SPSS for windows, version 11; was used for data analysis. All values were given as mean ± SD. Chi-square test and ANOVA were used in data analysis. Multiple comparison analysis by the least significant difference (LSD) was used to detect statistical difference between two means when ANOVA test referred to significances. The degree of relationship between the variables was calculated using Pearson correlation analysis for numerical data and Spearman correlation analysis for non numerical data. Data had not been transformed as data was normally distributed. Receiver operating characteristic (ROC) curve analysis was used to determine the discriminative properties of various cutoff levels of urinary hepcidin. A value of ≤ 0.05 was considered significant.

## Results

There were no significant differences between patients and control group as regards age, sex and bodyweight (P > 0.05) (Table 1). In our results, urinary hepcidin level in normal healthy control children was 2.8 ± 1.3 nmol/mmol Cr and the range was 0.62-6.65 nmol/mmol Cr.

**Table 1:** Clinical and laboratory data in patients and controls

	Control (n = 25)	(Stage-1 ID) (n = 25)	(Stage-2 ID) (n = 25)	(Stage-3 ID) (n = 25)	P
**Age **(year)	5.0 ± 0.9	5.1 ± 1.2	5.2 ± 1.3	4.9 ± 1.2	> 0.05

Male/female *	14/11	14/11	13/12	14/11	> 0.05

**Body weight **(kg)	17.2 ± 2.2	16.7 ± 2.3	16.0 ± 3.1	16.4 ± 2.6	> 0.05

**Hb **(g/dl)	12.5 ± 0.6^a^	11.9 ± 1.1^a^	11.8 ± 1.2^a^	7.9 ± 1.2^b^	< 0.05

**Hematocrit **(%)	37.6 ± 2.1^a^	35.8 ± 2.4^a^	35.5 ± 1.5^a^	23.6 ± 2.1^b^	< 0.05

**MCV **(fl)	92.3 ± 7.0^a^	87.8 ± 5.1^a^	86.8 ± 4.6^a^	61.1 ± 3.1^b^	< 0.05

**MCHC **(%)	34.1 ± 2.6^a^	34.0 ± 2.1^a^	33.9 ± 2.3^a^	26.1 ± 2.4^b^	< 0.05

**Serum iron **(ug/dl)	95.1 ± 4.6^a^	89.8 ± 2.5^a^	55.8 ± 2.5^b^	20.6 ± 1.1^c^	< 0.05

**TIBC**(mg/dl)	264 ± 15^a^	254 ± 34^a^	391 ± 24^b^	483 ± 21^c^	< 0.05

**Tsat **(%)	36.0 ± 1.1^a^	35.4 ± 0.6^a^	14.3 ± 0.9^b^	4.3 ± 0.6^c^	< 0.05

**Transferrin **(umol/L)	34 ± 2.3^a^	36 ± 2.5^a^	35 ± 3.4^a^	76.2 ± 13.6^b^	< 0.05

**Ferritin **(ng/ml)	69.2 ± 4.1^a^	16.0 ± 1.6^b^	10.5 ± 1.3^c^	5.3 ± 1.1^d^	< 0.05

**ZnPP **(ug/dl)	27 ± 1.3^a^	28 ± 2.3^a^	129 ± 10.1^b^	134 ± 12.3^b^	< 0.01

**Urinary hepcidin-25 **(nmol/mmol Cr)	2.8 ± 1.3^a^	0.7 ± 0.22^b^	0.3 ± 0.09^c^	.079 ± .009^d^	< 0.01

The P value is for the difference between all groups by ANOVA test. aa, bb = non-significant (p > 0.05). ab, ac, ad = significant (P < 0.05). bc, bd, cd = significant (P < 0.05). *Chi-square test. Tsat = Transferrin saturation, ZnPP = erythrocyte zinc protoporphyrin.

Urinary hepcidin levels were significantly lower in all stages of ID than in control group, more significant reduction in its level was observed with the progress in severity of ID (P < 0.01) (Table 1).

Receiver Operating Characteristics (ROC) curve was used to detect three cutoff points for urinary hepcidin level to differentiate ID, in its different stages, from healthy children. Cutoff points differentiating ID (stage-1, stage-2 and stage-3 respectively) from healthy children were ≤0.94, ≤0.42 and ≤0.08 nmol/mmol Cr respectively. Area under ROC curve was 0.838 (p < 0.001), 0.944 (p < 0.001) and 0.999 (p < 0.001) respectively. 95% confidence interval was (0.707-0.927), (0.840-0.988) and (0.927-1.000) respectively (Table 2). Sensitivity of these three cutoff points was 88%, 96% and 96% respectively. Specificity of these three cutoff points were 88%, 92% and 100% respectively. Positive predictive value of these three cutoff points was 88%, 92.3% and 100% respectively. Negative predictive value of these cutoff points were 88%, 95.8% and 96.2% respectively (Table 2).

**Table 2:** Predictive values of urinary hepcidin level in detection of iron deficiency

	Urinary hepcidin-25 level for detection of		
	**Stage-1(ID)**	**Stage-2(ID)**	**Stage-3(IDA)**

**Area under ROC curve**	0.838	0.944	0.999

**Standard error**	0.058	0.034	0.004

**95%confidence interval**	0.707-0.927	0.840-0.988	0.927-1.000

**Significance level p (area = 0.05)**	0.0001	0.0001	0.0001

**Cut off point (nmol/mmol Cr)**	≤ 0.94	≤ 0.42	≤ 0.08

**Sensitivity (%)**	88.0	96.0	96.0

**Specificity (%)**	88.0	92.0	100.0

**Positive predictive value (%)**	88.0	92.3	100

**Negative predictive value (%)**	**88.0**	**95.8**	**96.2**

**Figure 1:**
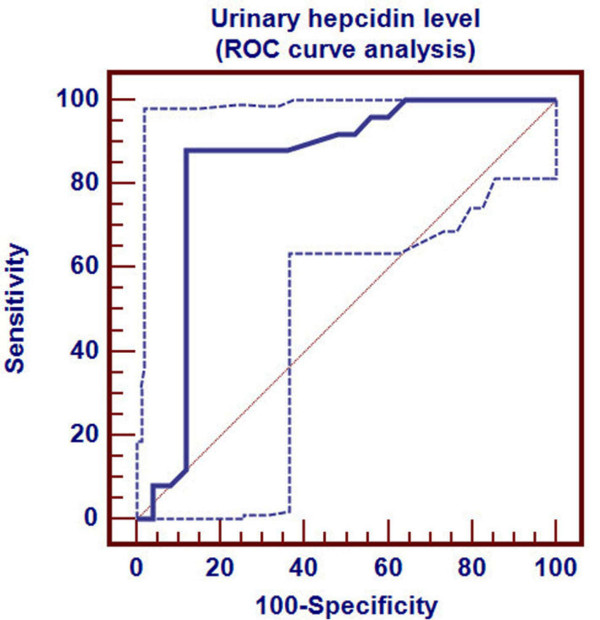
**Determination of hepcidin cutoff level for ID stage- 1**.

**Figure 2:**
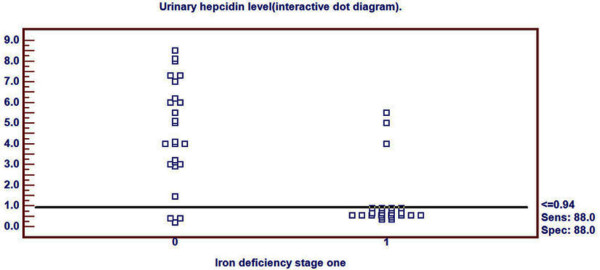
**Distribution of ID stage- 1 patients and controls around the cutoff point**.

Urinary hepcidin cutoff point for detection of ID stage-1 by ROC curve was demonstrated in Figure 1. Distribution of ID stage-1 patients and control group around this cutoff point was shown in Figure 2.

**Figure 3:**
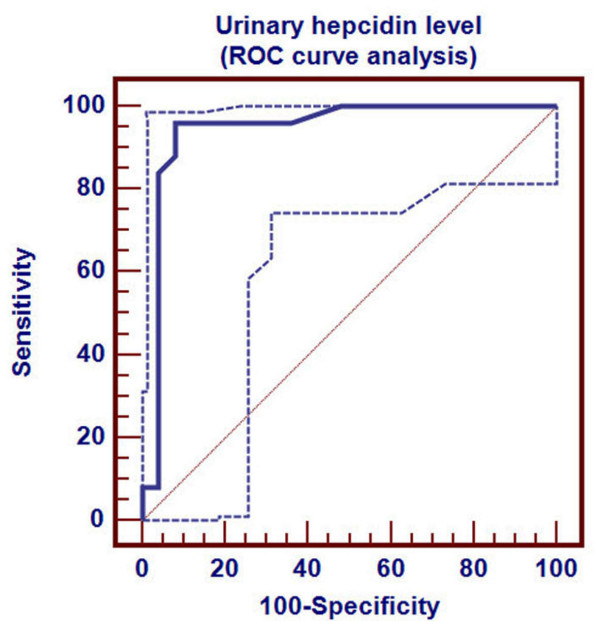
**Determination of hepcidin cutoff level for ID stage- 2**.

**Figure 4:**
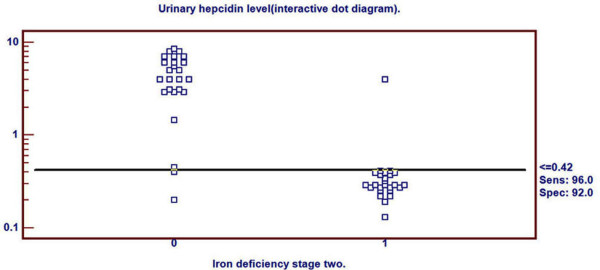
**Distribution of ID stage- 2 patients and controls around the cutoff point**.

Urinary hepcidin cutoff point for detection of ID stage-2 by ROC curve was demonstrated in Figure 3. Distribution of ID stage-2 patients and control group around this cutoff point was shown in Figure 4.

**Figure 5:**
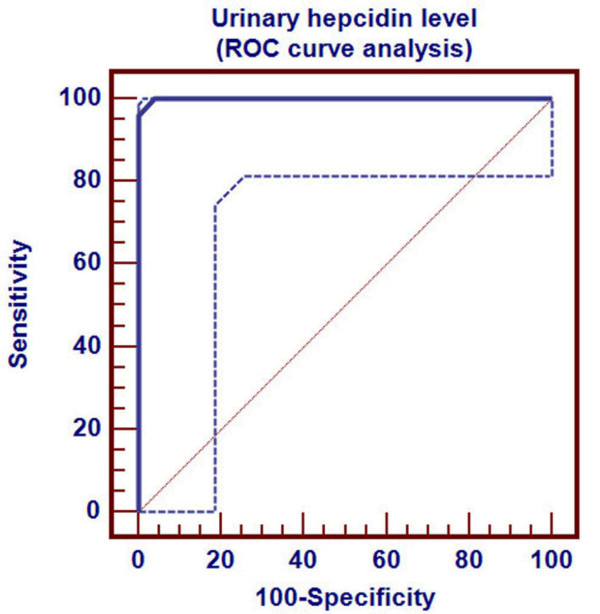
**Determination of hepcidin cutoff level for ID stage- 3**.

**Figure 6:**
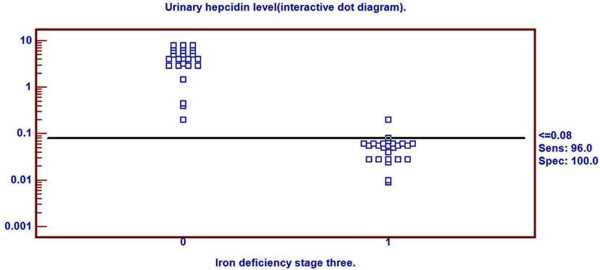
**Distribution of ID stage- 3 patients and controls around the cutoff point**.

Urinary hepcidin cutoff point for detection of ID stage-3 by ROC curve was demonstrated in Figure 5. Distribution of ID stage- 3 patients and control group around this cutoff point was shown in Figure 6.

Urinary levels of hepcidin showed significant positive correlation with Hb, hematocrit value, MCV, MCHC, serum iron level, ferritin level and Tsat (P < 0.01) (Table 3). In contrary, urinary levels of hepcidin showed significant negative correlation with serum transferrin and TIBC (P < 0.01) (Table 3).

**Table 3:** Pearson correlation between urinary hepcidin and clinico-laboratory parameters

	Urinary hepcidin-25 level	
	***r***	***p***

**Age **(year)	0.112	> 0.05

**Gender **(male/female)	0.125	> 0.05

**Body weight **(kg)	0.137	> 0.05

**Hb **(g/dl)	0.488	< 0.01

**Hematocrit **(%)	0.434	< 0.01

**MCV **(fl)	0.325	< 0.01

**MCHC **(%)	0.364	< 0.01

**Serum iron **(ug/dl)	0.378	< 0.01

**TIBC **(mg/dl)	-0.431	< 0.01

**Transferrin **(umol/L)	-0.398	< 0.01

**Transferrin saturation **(%)	0.306	< 0.01

**Ferritin **(ng/ml)	0.431	< 0.01

P > 0.05 = non significant, P < 0.05 = significant, P < 0.01 = highly significant.

## Discussion

The ideal screening test would be capable of identifying ID in the absence of anemia. This would help in the treatment of ID in the pre-anemic stage, preventing IDA and it's associated mental, motor and behavior effects. Such test is not widely used at this time [2].

In our study, urinary hepcidin levels were significantly lower in all stages of ID than in control group, more significant reduction in its level was observed with the progress in severity of ID. That coincided with Cherian et al et al [17] who found that urinary hepcidin levels were significantly lower in ID and IDA. Hepcidin is homeostatically regulated by iron and erythropoietic activity. Hepcidin is suppressed in ID, allowing increased absorption of dietary iron and replenishment of iron stores [15]. The feedback loop between iron and hepcidin ensures stability of plasma iron concentrations [30]. Hepcidin production is also regulated by the process which consumes most iron, erythropoiesis [23]. Increased erythropoietic activity suppresses hepcidin production which allows the release of stored iron from macrophages and hepatocytes, and increased iron absorption, all resulting in greater supply of iron for hemoglobin synthesis [22].

In our study, we determined three cutoff points for urinary hepcidin level to differentiate ID in its different stages (stage-1, stage-2 and stage-3) from healthy children. These three cutoff points had strong confidence intervals and valuable predictive potentials.

Guyatt et al [31] calculated the predictive value and area under the receiver operating characteristic (ROC) curve for serum ferritin in detection of IDA. Area under the receiver operating characteristic (ROC) was 0.95 (*p *< 0.001), compared to 0.77 for MCV, 0.74 for transferrin saturation, and 0.62 for absolute red cell distribution wideness (RDW).

In our study, urinary hepcidin levels at cutoff point ≤0.08 nmol/mmol Cr could predict ID stage-3 with Sensitivity 96% and specificity 100%. Furthers, urinary hepcidin levels at cutoff point ≤0.42 nmol/mmol Cr could predict ID stage-2 with sensitivity 96% and specificity 92%.

There is a shortage of iron available to the erythroid precursors in the bone marrow for hemoglobin synthesis in the second stage of ID [7]. The second stage may be characterized by abnormalities in particular iron parameters, including low Tsat and elevation in ZnPP level. Hemoglobin levels may be reduced but the resulting mild anemia may not be detectable using normal cutoff values for hemoglobin. Iron deficient erythropoiesis may be undetectable by using traditional laboratory parameters. In iron deficient erythropoiesis (second stage ID), storage iron may be normal or even increased due to impaired release of iron into the circulation [6].

In our study, urinary hepcidin levels at cutoff point ≤0.94 nmol/mmol Cr could predict ID stage-1 with sensitivity 88% and specificity 88%.

Beutler et al [5] stated that there is no overt effect on erythropoiesis in the first stage of ID; blood hemoglobin levels are usually normal, and ID generally can escape detection by hemoglobin or hematocrit screening. We obtained these relatively low values due to presence of three false positive cases and three false negative cases at the cutoff level ≤0.94 nmol/mmol Cr. We could not exactly explain if this result was due to fallacies in ferritin assay, which might be associated with missed cases (false negative) in control group and over estimation (false positive) in ID stage-1 group, or it might be related to limitation in hepcidin at this relatively high (≤0.94 nmol/mmol Cr) cutoff level. An explanation for our findings may stem from the elucidation of Kis et al [32] in a retrospective study of 101 patients, who had undergone bone marrow aspiration, as they found that a ferritin of ≤100 μg/l had 64.9% sensitivity and 96.1% specificity for IDA. It is noteworthy that ferritin level increases with age, and is an acute-phase reactant that may be falsely elevated in the setting of chronic inflammation, infection, malignancy and chronic renal failure [23,31]. In this situation, performing bone marrow aspiration may provide more explanation about this finding through estimation of stainable tissue iron.

In our study, urinary levels of hepcidin showed significant positive correlation with Hb, MCV, MCHC, hematocrit value, serum iron level, ferritin level and Tsat (P < 0.01). On the other hand urinary levels of hepcidin showed significant negative correlation with serum transferrin and TIBC (P < 0.01).

That agreed with the study which carried by Cherian et al [17], they demonstrated that hepcidin was positively associated with hemoglobin, MCV, iron, ferritin and Tsat levels. In contrary, hepcidin was negatively associated with transferrin.

One of our limitations in this study was the small number of cases as we tried to select demographic matched groups. To the best of our knowledge, this was the first trial to determine cutoff level for hepcidin in diagnosis of iron deficiency.

## Conclusions

Detection of urinary hepcidin-25 level was a simple and non invasive test and could predict iron deficiency very early, before appearance of hematological affection.

These findings raise the issue as to whether it would be beneficial to recommend urine hepcidin as a preliminary qualifying test for detection of very early iron deficiency in children and blood donors. A wider and larger multicenter prospective study will be necessary to confirm these findings in other populations.

## Competing interests

The authors declare that they have no competing interests.

## Authors' contributions

MS participated in the design, collected samples and also participated in the analysis of data and discussion. AG conceived of the study and participated in the design, coordinated the sample collection and reviewed the results and discussion. All authors read and approved all the manuscript.
